# Inhibition of adenylyl cyclase 1 by ST034307 inhibits IP_3_-evoked changes in sino-atrial node beat rate

**DOI:** 10.3389/fphar.2022.951897

**Published:** 2022-08-29

**Authors:** Samuel J. Bose, Matthew J. Read, Emily Akerman, Rebecca A. Capel, Thamali Ayagama, Angela Russell, Derek A. Terrar, Manuela Zaccolo, Rebecca A. B. Burton

**Affiliations:** ^1^ Department of Pharmacology, University of Oxford, Oxford, United Kingdom; ^2^ Department of Chemistry, Chemistry Research Laboratory, University of Oxford, Oxford, United Kingdom; ^3^ Department of Physiology, Anatomy and Genetics, University of Oxford, Oxford, United Kingdom

**Keywords:** inositol trisphosphate, adenylyl cyclase, cyclic AMP, cardiac atria, calcium signalling, pacemaking, sinoatrial node

## Abstract

Atrial arrhythmias, such as atrial fibrillation (AF), are a major mortality risk and a leading cause of stroke. The IP_3_ signalling pathway has been proposed as an atrial-specific target for AF therapy, and atrial IP_3_ signalling has been linked to the activation of calcium sensitive adenylyl cyclases AC1 and AC8. We investigated the involvement of AC1 in the response of intact mouse atrial tissue and isolated guinea pig atrial and sino-atrial node (SAN) cells to the α-adrenoceptor agonist phenylephrine (PE) using the selective AC1 inhibitor ST034307. The maximum rate change of spontaneously beating mouse right atrial tissue exposed to PE was reduced from 14.5% to 8.2% (*p* = 0.005) in the presence of 1 μM ST034307, whereas the increase in tension generated in paced left atrial tissue in the presence of PE was not inhibited by ST034307 (Control = 14.2%, ST034307 = 16.3%; *p* > 0.05). Experiments were performed using isolated guinea pig atrial and SAN cells loaded with Fluo-5F-AM to record changes in calcium transients (CaT) generated by 10 μM PE in the presence and absence of 1 μM ST034307. ST034307 significantly reduced the beating rate of SAN cells (0.34-fold decrease; *p* = 0.003) but did not inhibit changes in CaT amplitude in response to PE in atrial cells. The results presented here demonstrate pharmacologically the involvement of AC1 in the downstream response of atrial pacemaker activity to α-adrenoreceptor stimulation and IP_3_R calcium release.

## Introduction

Cardiac activity is closely regulated by the action of Ca^2+^ dependent enzymes including calcineurin and Ca^2+^/calmodulin-dependent kinase II (CaMKII), as well as Ca^2+^ mobilising agents such as inositol-1,4,5-trisphosphate (IP_3_), cyclic ADP-ribose (cADPR) and nicotinic acid adenine-dinucleotide phosphate (NAADP) (Bers, 2002; Terrar, 2020). In ventricular and atrial cardiomyocytes, calcium handling is key to the process of excitation contraction coupling (ECC), which is primarily regulated via the action of β-adrenergic signalling (Bers, 2002). Although a more modest component, ECC in the atria and sino-atrial node (SAN) is also regulated by α-adrenergic signalling (Lipp et al., 2000; Mackenzie et al., 2002; Capel et al., 2021). In addition to the regulation of ECC, currents that contribute to the pacemaker potential in the SAN and atrioventricular node (AVN) are also highly dependent on the regulation of intracellular Ca^2+^ signalling (Hancox and Mitcheson, 1997; Lakatta et al., 2010; Capel and Terrar, 2015; Burton and Terrar, 2021). In nodal cells, the pacemaker potential is dependent upon diastolic depolarisation, in part due to the influence of the hyperpolarisation activated “funny” current I_f_ and local calcium release events from the sarcoplasmic reticulum (SR). Depolarisation leads to Ca^2+^ influx through L-type Ca^2+^ channels (LTCC, principally Ca_v_1.3 in the SAN) subsequent Ca^2+^ release from the sarcoplasmic reticulum (SR) via ryanodine receptors (RyR), and activation of Na^+^/Ca^2+^ exchanger (NCX) (Lakatta et al., 2010; Tsutsui et al., 2018). In the atria and SAN, activation of the IP_3_ signalling pathway and subsequent release of Ca^2+^ from the SR may also lead to downstream activation of calcium sensitive adenylyl cyclase (AC), including isoforms AC1 and AC8 (Georget et al., 2002; Mattick et al., 2007; Burton and Terrar, 2021; Capel et al., 2021).

Under normal physiological conditions, both cardiac ECC and pacemaker activity are primarily regulated via β-adrenergic and muscarinic signalling, leading to downstream activation of adenylyl cyclase, predominantly AC5 and AC6 (Katsushika et al., 1992; Premont et al., 1992), and subsequent generation of cyclic-adenosine monophosphate (cAMP) (Bers, 2002; Burton and Terrar 2021). In ventricular cardiomyocytes, IP_3_ receptors (IP_3_R) are largely confined to the nuclear envelope and IP_3_ signalling is not thought to play a major role in cellular Ca^2+^ handling (Nakayama et al., 2010; Tinker et al., 2016). However, IP_3_ is thought to play a greater role in Ca^2+^ handling downstream of α-adrenergic signalling within the atria, and atrial cardiomyocytes show a higher overall expression of IP_3_R with expression in the subsarcolemmal space (Lipp et al., 2000; Mackenzie et al., 2002; Tinker et al., 2016). Whilst the role of β-adrenergic signalling in the regulation of ECC and pacemaker activity is relatively well studied, less is known about the potential involvement of α-adrenergic activation, leading to the generation of IP_3_ and diacylglycerol (DAG) *via* activation of protein kinase C (PKC) in the regulation of these processes in the atria and SAN. Increasing evidence indicates that activation of IP_3_R and subsequent Ca^2+^ release from SR, leads to downstream activation of adenylyl cyclase, potentially via the activation of calcium-sensitive isoforms AC1 and AC8 (Domeier et al., 2008; Terrar, 2020; Burton and Terrar, 2021; Capel et al., 2021). In the present study, we therefore chose to focus on investigating the downstream effects of α-adrenergic stimulation in these processes.

### The role of AC1 in cardiac pacemaker cells

AC1 is preferentially expressed in the SAN compared to other regions of the heart, and is thought to play a role in the regulation of pacemaker activity via modulation of the hyperpolarisation activated “funny” current (I_f_) (Mattick et al., 2007; Younes et al., 2008). In the SAN, spontaneous pacemaker activity is the result of the “coupled-clock” mechanism, involving tight coupling between rhythmic Ca^2+^ release from the SR (i.e., “Ca^2+^ clock”), and rhythmic oscillations in the membrane potential (i.e., “membrane clock”) (Lakatta et al., 2010; Tsutsui et al., 2018). Indeed, it appears this coupling is essential for pacemaker function in human SAN cells (Tsutsui et al., 2018). Membrane clock activity results from the alternation and balance between depolarising currents (e.g., I_f_, I_CaL_, I_sust_) and repolarising currents (e.g., I_Ks_ and I_Kr_) (Difrancesco and Tromba, 1988; Difrancesco et al., 1991; Burton and Terrar, 2021). I_f_ is carried by the HCN channels and modulated by changes in cytosolic Ca^2+^ (Rigg et al., 2003), as well as sub-sarcolemmal cAMP (Difrancesco and Tortora, 1991; Burton and Terrar, 2021). I_f_, and therefore SAN pacemaking, can thereby be influenced by phosphodiesterase (PDE) and AC activity (Difrancesco and Tortora, 1991; Mattick et al., 2007; Vinogradova et al., 2008).

AC1 activity modulates the I_f_ current in the SAN in the absence of β-adrenergic stimulation and contributes to the higher resting cAMP level in SAN cells compared to ventricular cells (Mattick et al., 2007; Younes et al., 2008). In addition, cAMP generated by AC1 may modulate RyR, SR Ca^2+^-ATPase, NCX and LTCC, all of which are involved in determining spontaneous beating in the SAN (Younes et al., 2008). These observations suggest cAMP signalling, downstream of AC1 activation, is a crucial mechanism by which the Ca^2+^ clock and membrane clock are coupled in the SAN. Furthermore, the modulation of I_f_ by cytosolic Ca^2+^ appears to be independent of CaMKII as chelation of SAN Ca^2+^ using BAPTA reduces I_f_, whereas inhibition of CaMKII is without effect (Rigg et al., 2003). Although CaMKII is essential for SAN pacemaker activity (Yaniv et al., 2013), the actions of CaMKII on pacemaker function are linked to effects on I_Ca,L_ rather than I_f_ (Vinogradova et al., 2000; Rigg et al., 2003). Conversely, inhibition of SAN ACs using the non-specific AC inhibitor MDL-12,330A reduces I_f_, whilst inhibition of phosphodiesterase using IBMX to inhibit the breakdown of basal cAMP increases I_f_, suggesting a role for Ca^2+^-activated ACs in regulating the I_f_ current in SAN cells (Mattick et al., 2007).

### AC1 expression in atrial cardiomyocytes

In guinea pig atrial cardiomyocytes, AC1 and AC8 are localised in the plasma membrane in close proximity to type 2 IP_3_ receptors (IP_3_R2) on the SR (Capel et al., 2021). Non-specific inhibition of ACs using MDL-12,330A, or inhibition of PKA using H89, inhibits the increase in Ca^2+^ transient amplitude observed in isolated guinea pig atrial cardiomyocytes in response to either intracellular photorelease of caged IP_3_ (IP_3_/PM) or external stimulation of α-adrenergic signalling using phenylephrine (PE), thereby demonstrating that AC’s can be activated downstream of IP_3_R. The increase in spontaneous beating rate observed in intact murine right atria in response to PE is similarly inhibited using either MDL-12,330A or H89 (Capel et al., 2021), suggesting a role for Ca^2+^-activated AC1 and or AC8 in the positive inotropic response to IP_3_ signalling in cardiac atria.

Given the potential role of AC1 in regulating the downstream effects of α-adrenergic signalling in both atrial cardiomyocytes and the SAN, we were interested in investigating the pharmacological modulation of AC1 in cardiac tissue. The development of small molecule AC1 inhibitors that are highly selective for AC1 over AC8 has been of interest for treatment of neuropathic and inflammatory pain, leading to the development of the compounds such as NB001 (Wang et al., 2011) and the chromone derivative ST034307 (Brust et al., 2017). In this study, we chose to investigate whether pharmacological inhibition of cardiac AC1 by ST034307 could affect the response to α-adrenergic signalling in both intact atrial tissue as well as isolated SAN cells.

## Materials and methods

### Animals

All animal experiments were performed in accordance with the United Kingdom Home Office Guide on the Operation of Animal (Scientific Procedures) Act of 1986. All experimental protocols (Schedule 1) were approved by the University of Oxford, Procedures Establishment License (PEL) Number XEC303F12.

### Drugs and reagents

AC1 was inhibited using the AC1 selective inhibitor ST034307 (Tocris, United Kingdom), which has been shown to demonstrate selectivity for AC1 over other AC isoforms at concentrations below 30 μM (Brust et al., 2017). In all experiments, ST034307 was dissolved in DMSO to make 3 mM stock prior to addition to experimental solutions at a final concentration of 1 µM and applied for at least 5 min for isolated cells, or 30 min for whole-tissue experiments in order to ensure sufficient tissue penetration.

### Atrial myocyte isolation

Male Dunkin Hartley guinea pigs (300–550 g, Envigo, United Kingdom) were housed and maintained in a 12 h light-dark cycle with *ad libitum* access to standard diet and sterilized water. Guinea pigs were culled by concussion followed by cervical dislocation in accordance with Home Office Guidance on the Animals (Scientific Procedures) Act (1986). Atrial myocytes were isolated following the method of Collins et al. (2011). Hearts were rapidly excised and washed in physiological salt solution (PSS, in mM): NaCl 125, NaHCO_3_ 25, KCl 5.4, NaH_2_PO_4_ 1.2, MgCl_2_ 1, glucose 5.5, CaCl_2_ 1.8, oxygenated with 95% O_2_/5% CO_2_ (solution pH 7.4 after oxygenation and heating) to which heparin was added (final concentration = 20 IU·ml^−1^) to prevent clot formation in the coronary vessels. Hearts were then mounted on a Langendorff apparatus for retrograde perfusion via the aorta. Perfusion was initially carried out in a modified Tyrode solution containing (mM): NaCl 136, KCl 5.4, NaHCO_3_ 12, Na^+^ pyruvate 1, NaH_2_PO_4_ 1, MgCl_2_ 1, EGTA 0.04, glucose 5; gassed with 95% O_2_/5% CO_2_ to maintain a pH of 7.4 at 35 ± 1°C. After 2 min this was replaced with a digestion solution: the modified Tyrode above containing 100 µM CaCl_2_ and either 0.6 mg/ml of collagenase (type II, Worthington Biochemical Corp., Lakewood, NJ, United States) or 0.02–0.04 mg/ml Liberase™ TH (Roche, Penzberg, Germany), but no EGTA.

After this enzymatic digestion, the heart was removed from the cannula and the atria were separated from the ventricles. For the isolation of atrial myocytes, slices of the atria were triturated using a glass pipette and stored at 4°C in a high potassium medium containing (in mM): KCl 70, MgCl_2_ 5, K^+^ glutamine 5, taurine 20, EGTA 0.1, succinic acid 5, KH_2_PO_4_ 20, HEPES 5, glucose 10; pH to 7.2 with KOH. For the isolation of SAN cells, the translucent SAN region, located on the upper surface of the right atrium, in between the inferior and superior vena cava (Rigg et al., 2003), immediately medial to the crista terminalis was identified under a dissection microscope. Thin tissue strips encompassing the nodal region were dissected, triturated using a glass pipette and stored at 4°C in high potassium medium. For experiments, healthy atrial myocytes were identified based on morphology, and healthy SAN myocytes by morphology and the presence of spontaneous, rhythmic beating in the absence of electrical stimulation.

### Murine atrial studies

Adult male CD1 mice (30–35 g, Charles River, United Kingdom) were housed in a 12 h light-dark cycle with *ad libitum* access to standard diet and sterilized water. Mice were culled by concussion followed by cervical dislocation in accordance with Home Office Guidance on the Animals (Scientific Procedures) Act (1986). The heart was rapidly excised and washed in heparin-containing PSS. The ventricles were dissected away under a microscope and the atria were cleared of connective tissue before being separated. Right and left atrial preparations were mounted separately in a 37°C organ bath containing oxygenated PSS and connected to a force transducer (MLT0201 series, ADInstruments, United Kingdom) in order to visualize contractions. Resting tension was set between 0.2 and 0.3 g, and the tension signals were low-pass filtered (20 Hz for right atria and 25 Hz for left atria). Right atrial beating rate was calculated from the time interval between contractions. Left atria were electrically field stimulated at a constant rate of 5 Hz using a custom-built stimulator connected to coil electrodes positioned both sides lateral to the left atrial tissue. Voltage was set at the threshold for stimulating contraction plus 5 V, and was within the range 10–20 V for all experiments. In all experiments, preparations were allowed to stabilise at a resting beating rate (>300 bpm) in PSS for at least 30 min. After stabilisation (variation in average rate of a 10 s sample of no more than 2 bpm over a 10-min period or 0.01 g change in tension), metoprolol (1 µM) was added to the bath to ensure specificity to α-adrenergic effects, plus or minus ST034307 (1 µM). Each addition was allowed to stabilise for a further 30 min or until stability was achieved as described above. Cumulative concentrations of PE were added to the bath at intervals of 5 min (range 0.1–30 µM) in the presence of metoprolol. Preparations were excluded if stabilized beating rate under control conditions (PSS only) was less than 300 bpm, in the case of the right atrium, or if preparations were not rhythmic. Data were fitted using Log(agonist) versus response curves (three parameter model) by nonlinear regression using a least squares method (Prism v9). AC1 was inhibited using the AC1 selective inhibitor ST034307 (Tocris, United Kingdom) (Brust et al., 2017). ST034307 (1 µM) was added after stabilization of the preparations in the presence of metoprolol and applied for at least 30 min prior to PE additions. PE dose-response curves were started only after tissue had reached a stable response.

### Immunocytochemistry

Immunocytochemical labelling and analysis was carried out using the method of Collins and Terrar (2012). Rabbit anti-AC1 (55067-1-AP) primary antibody was purchased commercially (ProteinTech, Manchester, United Kingdom) and used at a dilution of 1:200. Mouse anti-IP_3_R monoclonal primary antibody IP_3_R2 (sc-398434) was purchased commercially (Santa Cruz Biotechnology, Santa Cruz, CA, United States) and used at a dilution of 1:50. The specificity of antibody sc-398434 has been previously verified using Western blot by Lou et al. (2021). IP_3_R antibodies have been extensively covered in previous studies (Hattori et al., 2004; Ando et al., 2006; Uchida et al., 2010; Salvador and Egger, 2018). Isolated cardiac cells were plated onto flamed coverslips and left to adhere for 30 min at 4°C. Cells were first fixed in 4% paraformaldehyde–phosphate buffered saline (PBS) for 15 min. Once the cells were fixed, they were washed in PBS (3 changes, 5 min each). Cells were then permeabilised and blocked using the detergent Triton X-100 (0.1%), 10% horse serum and 10% BSA in PBS (Sigma-Aldrich) for 60 min at room temperature to reduce non-specific binding. After blocking, the cells were incubated with primary antibodies at 4°C overnight dissolved in blocking solution. The next day, cells were first washed with PBS (3 changes, 5 min each) before being incubated with secondary antibodies; AlexaFluor -488 or -546 conjugated secondary antibodies (Invitrogen, United Kingdom), raised against the appropriate species, at room temperature for 120 min in PBS then washed with PBS (3 changes, 5 min each). Finally, the cells were mounted using Vectashield with DAPI and permanently sealed with nail polish. Control cells where the primary or secondary was to be excluded were incubated with 10% horse serum and 10% BSA in PBS without addition of the relevant antibody.

Cells were stored in the dark at 4°C before imaging. Observations were carried out using a Nikon eclipse Ti inverted confocal microscope (Nikon) with a 63x/1.2 water objective Plan Apo VC 60xA WI DIC N2 lens. NIS-Element viewer (Nikon) was used to acquire multichannel fluorescence images. For detection of DAPI, fluorescence excitation was at 405 nm with emission collected at 450 nm. For detection of AlexaFluor 488, fluorescence excitation was at 488 nm with emission collected at 525 nm. Excitation at 561 nm was collected at 595 nm for detection of AlexaFluor 568. The two channels were imaged sequentially at 2048 × 2048 (12 bits). Z-stack images were collected at 2.112 µm sections.

### Ca^2+^ transient imaging

For whole-cell fluorescence experiments, isolated atrial myocytes were incubated with membrane permeant Fluo-5F-AM (3 µM) for 10 min then plated to a glass cover slip for imaging. Cells were incubated for a further 10 min *in-situ* in the organ bath to allow time for cells to adhere to the cover slip before perfusion with PSS. Carbon fibre electrodes were used to field-stimulate Ca^2+^ transients at a rate of 1 Hz. All experiments were carried out at 35 ± 2°C (fluctuation within a single experiment was <0.5°C) under gravity-fed superfusion with PSS, oxygenated with 95% O_2_/5% CO_2_ (solution pH 7.4 after oxygenation and heating). Solution flow rate was 3 ml min^−1^. Cells were visualized using a Zeiss Axiovert 200 with attached Nipkow spinning disc confocal unit (CSU-10, Yokogawa Electric Corporation, Japan). Excitation light, transmitted through the CSU-10, was provided by a 488 nm diode laser (Vortran Laser Technology Inc., Sacramento, CA, United States). Emitted light was passed through the CSU-10 and collected by an Andor iXON897 EM-CCD camera (Oxford Instruments, United Kingdom) recorded at a minimum acquisition frame rate of 60 frames per second using µManager software (v2.0) and ImageJ (Exposure time = 3–10 ms; binning = 4 × 4). In order to avoid dye bleaching the cells were not continually exposed to 488 nm light. Instead, a video of 8–10 s of Ca^2+^ transients was recorded at discrete timepoints. Ca^2+^ transient time courses were analysed in ImageJ and ClampFit (version 10.4). For analysis of Ca^2+^ transient rise and decay times Ca^2+^ data were analysed using pClamp v10 (Molecular Devices, CA, United States) to generate times corresponding to 10%–90% and 10%–50% rise time, and 90%–10%, 90%–75%, 90%–50% and half width decay time. Decay phases of transients were also fitted using one phase decay least squares regression (Prism v9).

### Statistics

Data were tested for normality by using a Shapiro-Wilk test in Prism v9 software (GraphPad, CA, United States). For all single cell data, two-way t-tests, repeated measures 2-way ANOVA or mixed effects analysis were used as appropriate, with Dunnett’s or Tukey’s post hoc test to compare groups to a single control or to all other groups as required (alpha = 0.05). For SAN data, Sidak’s multiple comparison was used to compare control and ST034307 data at all time points. Log[concentration]-response curves, used to estimate EC50s and maximum responses, were calculated using Prism v9 software (GraphPad, CA, United States), by fitting an agonist-response curve with a fixed slope to normalized response data. Normalized data was used to compare responses. Fitted values were compared using 2-way repeated measures ANOVA followed by Šídák’s multiple comparisons or Fisher’s LSD test. Data are presented as mean ± SEM of recorded values, other than dose-response curve maximums and EC50 which are given as mean ±95% confidence interval of best-fit value.

## Results

### Inhibition of AC1 by ST034307 reduces the positive chronotropic effect of PE in the intact sino-atrial node

**FIGURE 1:**
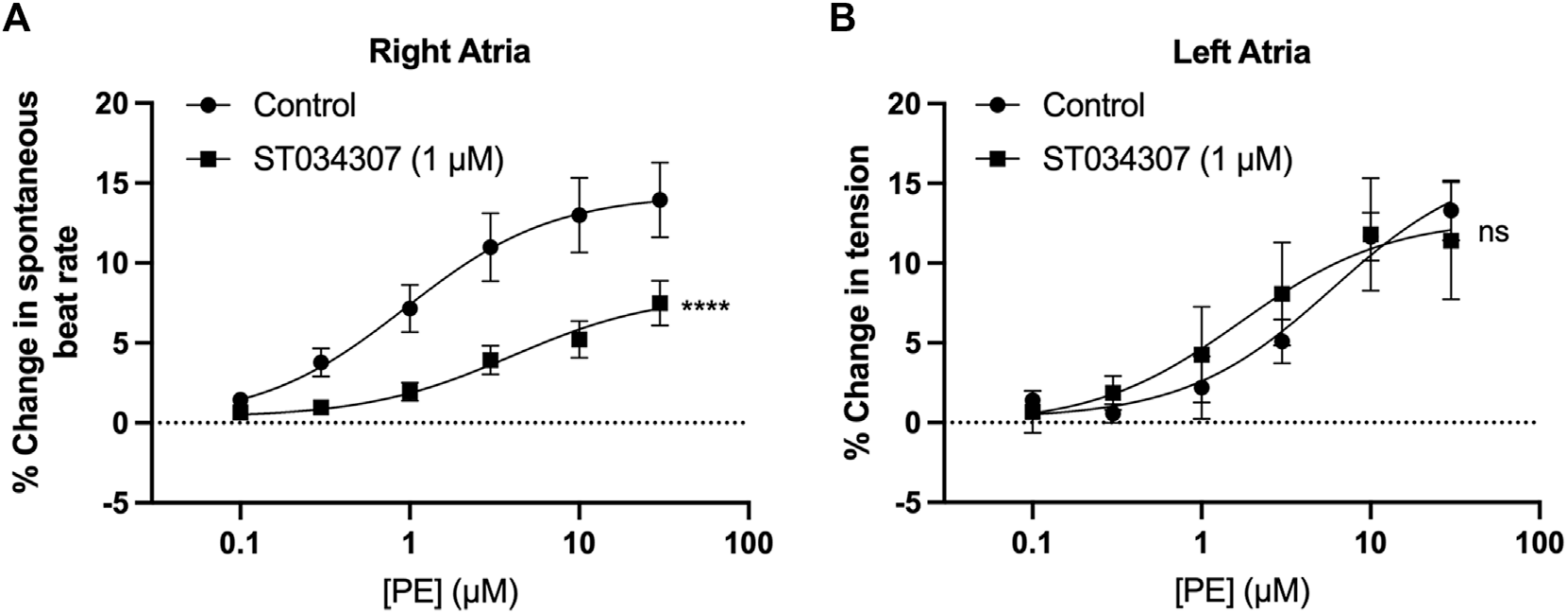
1 µM ST034307 inhibits changes in chronotropy in intact mouse right atria with intact SAN but not inotropy in left atria. **(A)** Dose response curves to show the change in beating rate on cumulative addition of PE to spontaneously beating mouse right atria preparations under control conditions (circles, *n* = 15) and in the presence of 1 µM ST034307 (squares, *n* = 12). **(B)** Dose response curves to show the change in tension generated on cumulative addition of PE to mouse left atria preparations under control conditions (circles, *n* = 5) and in the presence of 1 µM ST034307 (squares, *n* = 7). Dose-response curves in **(A,B)** (solid lines) were fitted using log(agonist) vs. response (three-parameter model) using Graphpad Prism v9. Asterisks indicate significance level for effect of ST034307 compared to control at individual concentrations as determined using 2-way repeated measures ANOVA followed by Šídák’s multiple comparisons test. Data are represented as mean ± SEM; *ns* = not significant; *, *p* < 0.05; **, *p* < 0.01; ***, *p* < 0.001.

Spontaneously beating right atrial tissue preparations, which contain the intact SAN, can be used to indirectly record activity of the SAN through the measurement of beating rate using a force transducer (Capel et al., 2015; Macdonald, 2020; Capel et al., 2021), whilst intact left atria can be used to record changes in contractile force generated when stimulated at a constant rate and voltage (Capel et al., 2015; Capel et al., 2021). Inhibition of ACs has previously been shown to reduce the response of intact mouse right atria to α-adrenergic stimulation (Capel et al., 2021). We were therefore interested to see if the effect of ST034307 was specific to the right atria, or whether the inotropic effects of PE would be inhibited by ST034307 in left atrial preparations. Isolated murine right and left atria were mounted separately in organ baths and perfused with PSS at 37°C in the presence of 1.0 µM metoprolol to inhibit β-adrenergic signalling. Dose response curves for either spontaneous beating rate (right atria, Figure 1A) or tension generated (left atria, Figure 1B) were generated in response to 0.1–30 μM PE.

In the absence of ST034307 but in the presence of metoprolol, the spontaneous beating rate of right atria increased by a maximum of 14.5% (95% confidence interval (CI) = 12.2–17.1%; *n* = 15) at 30 μM PE, with an EC50 of 0.9 µM (95% CI = 0.4–2.2 µM) (Figure 1A, round symbols). 1 µM ST034307 reduced the response of beating rate to PE at all concentrations tested (Figure 1A, square symbols), with a maximum increase of 8.2% (95% CI = 6.1–12.6%; *n* = 12) at 30 μM PE (EC50 = 3.9 µM; 95% CI = 1.1–17.6 µM). This represented a significant overall reduction in the response to PE in the presence of ST034307 (*p* = 0.005, 2-way repeated measures ANOVA, PE vs. ST034307). For left atrial preparations (Figure 1B), PE increased the tension generated in response to electrical stimulation at 5 Hz. The maximum increase was 14.2% (95% CI = 11.2–18.5%; *n* = 5) at 30 μM PE, with an EC50 of 4.4 µM (95% CI = 1.92–18.5 µM). In the presence of ST034307, no difference was observed in the response to PE compared with control (*p* > 0.05, 2-way repeated measures ANOVA, PE vs ST034307). The maximum change in tension generated in the presence of ST034307 was 16.3% (95% CI = 11.7–23.3; *n* = 7) at 30 μM PE, with an EC50 of 0.9 µM (95% CI = 0.8–10.8 µM). The basal heart rate of right atria after stabilisation and before addition of metoprolol was 364 ± 15.5 bpm (*n* = 12). No change in the right atrial basal heart rate was observed on addition of either metoprolol (362 ± 16.3 bpm) or metoprolol plus ST034307 (359 ± 16.8 bpm) before addition of PE (Supplementary Figure S1).

### Immunohistochemistry suggests IP_3_R2 and AC1 are colocalised in isolated Guinea pig atrial and sino-atrial node myocytes

**FIGURE 2:**
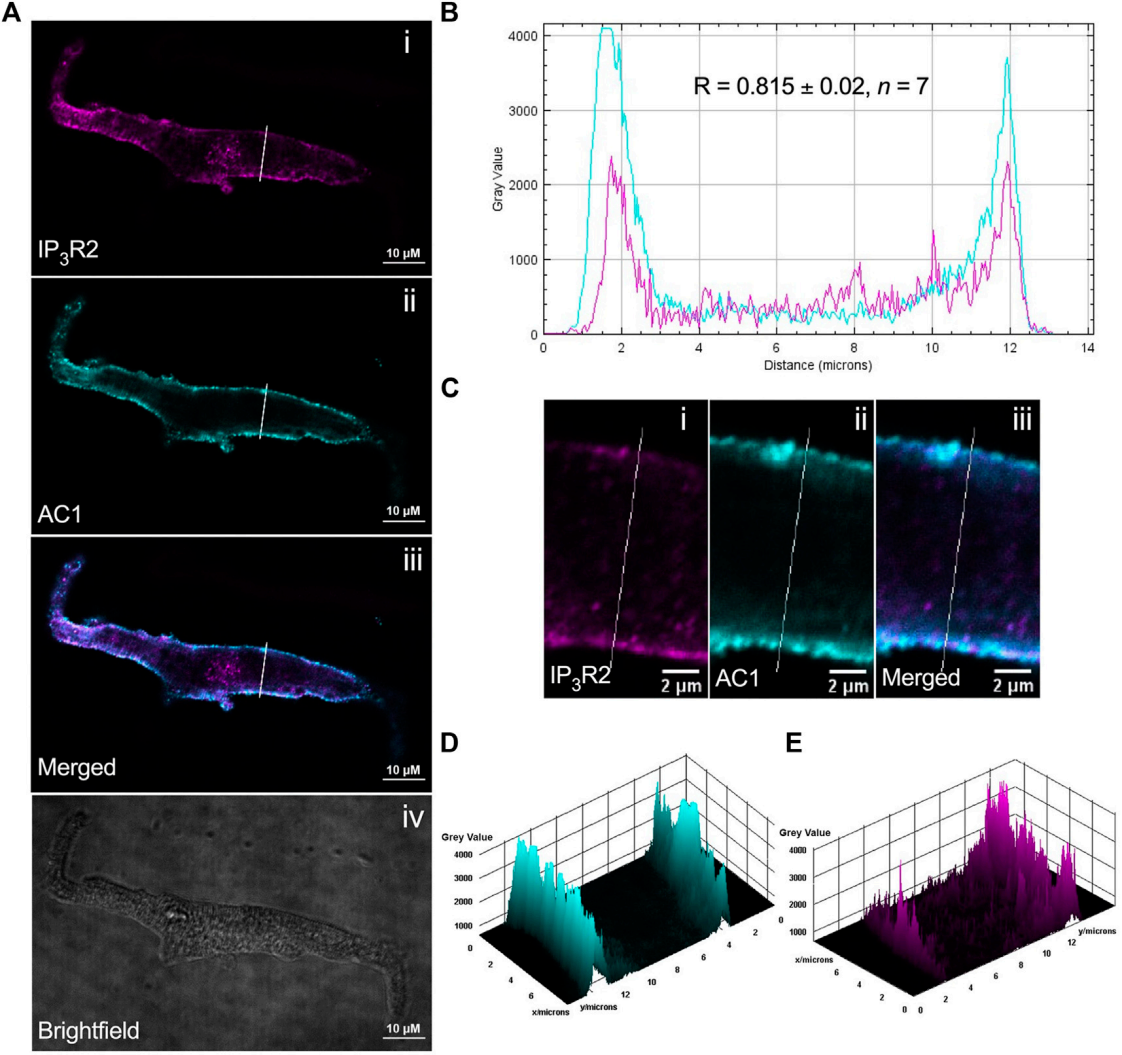
IP_3_R2 is expressed in close proximity with AC1 in guinea pig SAN myocytes. **(A)** Representative example of a fixed, isolated guinea pig SAN myocyte immunolabelled for (i) IP_3_R2 (magenta), (ii) AC1 (cyan), (iii) co-immunolabelled for IP_3_R2 (magenta) and AC1 (cyan). Example brightfield image shown in (iv). **(B)** Intensity plot to show staining intensity along the white line shown in **(A)**. R-value calculated from *n* = 7 cells. **(C–E)** Intensity surface plots showing the distribution of staining of IP_3_R2 [magenta, **(D)**] and AC1 [cyan, **(E)**] for the regions of cell shown in **(C)**. Scale bars representing 10 µm are indicated in **(A)** and 2 µm in **(C)**. For the purposes of presentation only, red and green channels have been represented as magenta and cyan, respectively.

ST034307 inhibited the beating rate response of the intact SAN (right atrial preparations) but did not alter tension generation in the left atria in response to PE. This suggested that the effects of AC1 inhibition were limited to affecting chronotropy in the SAN but not inotropy in atrial tissue (Figure 1). We were interested in testing whether the pattern of AC1 and IP_3_R2 in the SAN matched that observed in atrial cardiomyocytes reported previously (Capel et al., 2021). To determine structural and anatomical characterisation of AC1 and IP_3_R2 in SAN myocytes from healthy guinea pig adult hearts, isolated SAN myocytes were fixed and immunolabelled for AC1 and IP_3_R2. Figure 2 shows representative confocal images of SAN myocytes stained with primary antibodies raised against the AC1 (cyan) and IP3R (magenta) proteins. IP_3_R2 showed the expected staining on the sarcoplasmic reticulum membrane in SAN myocytes (Figure 2Ai) and AC1 puncta are located in close proximity to IP_3_R2 (Figure 2Aiii, pixel size = 0.05 × 0.05 µm). ImageJ intensity analysis revealed, pixel by pixel by line intensity plots (Figures 2A,B) and in whole image intensity plots (Figures 2C–E), levels of colocalization between AC1 and IP_3_R2 in SAN cells were higher (R = 0.82 ± 0.02, *n* = 7) compared to atrial myocytes (Supplementary Figure S2, R = 0.69 ± 0.02, *n* = 11, *p* < 0.001) and reported by Capel et al (2021) (*R* = 0.5 ± 0.05 *n* = 5)*.* These results together with recently published data (Capel et al., 2021), suggest that IP_3_R-dependent signalling may be capable of stimulating Ca^2+^-dependent ACs and the close positioning of AC1 to IP_3_R2 suggests that AC1 may be an effector of this interaction. To determine any non-specific labelling of secondary antibodies, control cells with no primary antibody, incubated with AlexaFluor -488 or -546 conjugated secondary antibodies alone, were imaged and found to give no detectable signal (data not shown).

### Inhibition of AC1 by ST034307 does not alter Ca^2+^ transient amplitude in isolated Guinea pig atrial myocytes, but does inhibit pacemaker activity in isolated sino-atrial node myocytes

To further investigate the differences observed between SAN and left atria in Figure 1, and to determine if the effect of AC1 inhibition by ST034307 is specific to the SAN, we investigated the effect of ST034307 on calcium transients generated in isolated guinea pig right atrial and SAN cells. We chose to investigate calcium transients in guinea pig cells, rather than mouse as guinea pig cardiac electrophysiology more closely resembles that of human. Whilst mouse heart physiology is comparable to human at the level of the whole heart, at the cellular level, guinea pig hearts share a more comparable action potential profile and heart rate to human and so is more appropriate for the study of isolated cells (O’Hara and Rudy, 2012).

### Effect of ST034307 on calcium transients stimulated Guinea pig atrial cells

**FIGURE 3:**
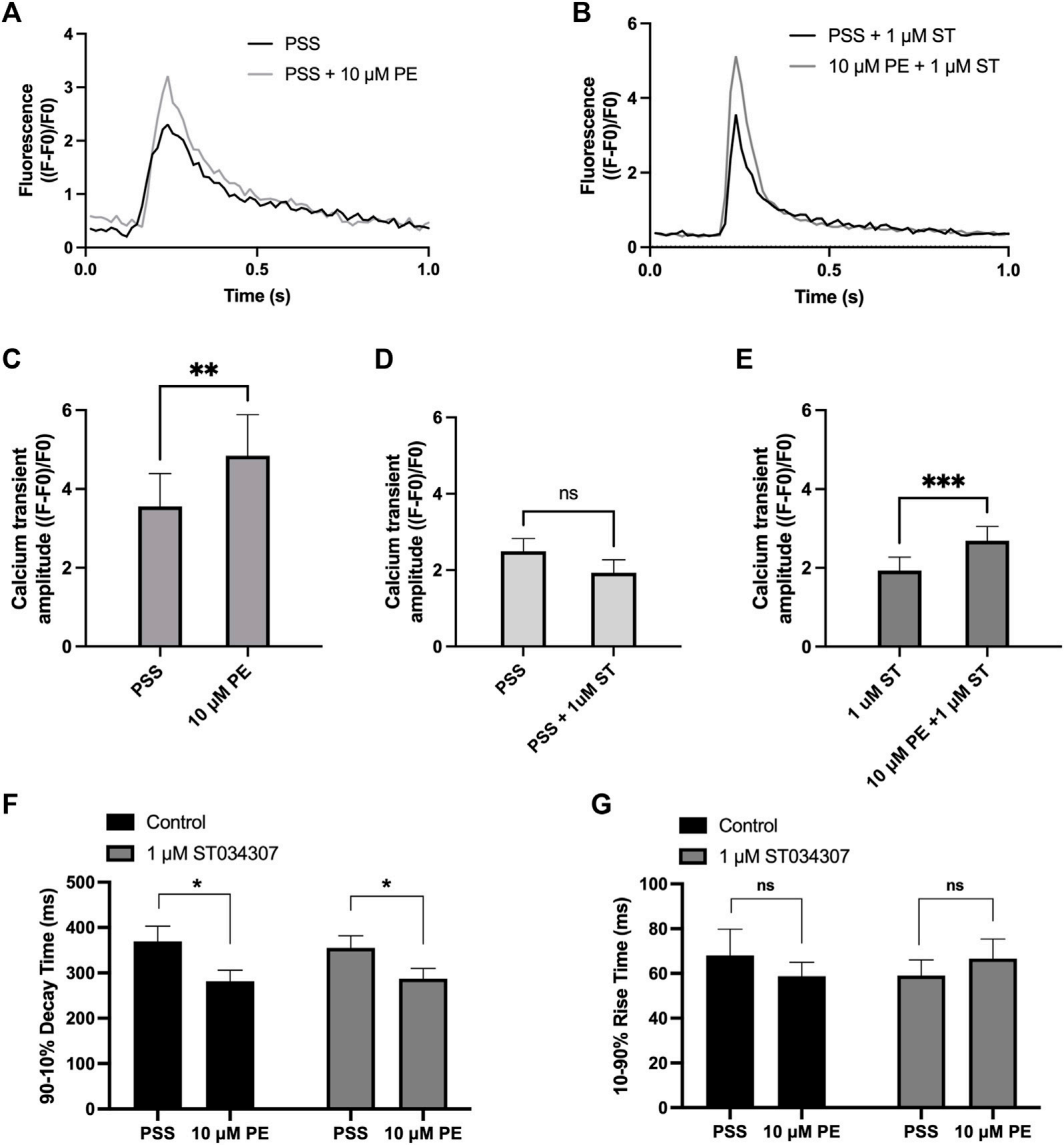
1 μM ST034307 does not alter the effect of PE on Ca^2+^ transients in isolated guinea pig atrial myocytes. **(A,B)**: Representative average Ca^2+^ transients recoded from atrial myocytes during baseline recording (black trace) and following addition of PE (grey trace) in the absence (B) or presence (C) of 1 μM ST034307. **(C)**: Effect of 10 μM PE on the Ca^2+^ transient amplitude recorded from isolated guinea pig atrial cells (*n* = 6; *N* = 3). **(D)**: Effect of 1 μM ST034307 on the basal Ca^2+^ transient amplitude before addition of PE (PE, *n* = 18; ST, *n* = 15; *N* = 3). **(E)**: Effect of 10 μM PE on the Ca^2+^ transient amplitude of cells in the presence of 1 μM ST034307 (*n* = 15; *N* = 3). **(F,G)**: Effect of 10 μM PE on the 90–10% decay time **(F)** and 10–90% rise time **(G)** of Ca^2+^ transients recorded from cells perfused with PSS in the absence (black bars) or presence (grey bars) of 1 μM ST034307 before and after addition of 10 μM PE. All experiments were carried out at 35 ± 2°C and recordings were made 5 min following the start of each solution perfusion. Data are represented as mean ± SEM. Data in C and E were analysed using paired t-test. Data in D were analysed using unpaired t-test. Data in F and G were analysed using two-way, repeated measures ANOVA followed by Šídák’s multiple comparisons test; ns = not significant; *, *p* < 0.05; **, *p* < 0.01; ***, *p* < 0.001; *n* = cells; *N* = animals.

To measure changes in cytosolic Ca^2+^ in response to PE, isolated guinea pig right atrial myocytes were loaded with the cell-permeant Ca^2+^ sensitive dye Fluo-5F-AM. When stimulated at 1 Hz in PSS at 37°C, atrial myocytes exhibited the classical pattern of Ca^2+^ transient observed previously (Huser et al., 1996; Capel et al., 2021). We expressed calcium transient amplitude as change in mean cell fluorescence (F-F0)/F0 (Figures 3A-E). Addition of PE (10 µM) to the perfusion solution resulted in a 0.4-fold increase in Ca^2+^ transient amplitude from 3.6 ± 0.8 to 4.8 ± 1.0 (*p* = 0.009; *n* = 6; paired t-test) (Figures 3A,C). As shown in Figure 3D, addition of 1 µM ST034307 did not alter the basal Ca^2+^ transient amplitude compared to perfusion with PSS alone (PSS = 2.5 ± 0.3, *n* = 18; 1 µM ST = 1.9 ± 0.3, *n* = 15; *p* > 0.05, unpaired t-test). In the presence of 1 μM ST034307, a 0.4-fold increase in the Ca^2+^ transient amplitude was observed in response to 10 μM PE added to the perfusion solution, resulting in a Ca^2+^ transient amplitude of 2.7 ± 0.4 (*p* = 6.0 × 10^−4^; *n* = 15) (Figures 3B,E). Comparison of the percentage change in response to PE between control and 1 µM ST034307 confirmed that there was no significant difference between the amplitude of change in the presence of ST (*p* > 0.05, unpaired t-test).

Further analysis of Ca^2+^ transient rise time and decay times confirmed that PE resulted in a significant decrease in the 90%–10% decay time in control experiments from 369.5 ± 33.5 ms to 281.3 ± 24.8 ms (*p* = 0.02, 2-way ANOVA, *n* = 8) (Figure 3F, black bars) without changing the 10–90% rise time (Figure 3G, black bars). In the presence of 1 μM ST034307, the same pattern was also observed with a decrease in 90%–10% decay time from 355.0 ± 26.8 ms to 287.4 ± 22.5 ms (*p* = 0.01, 2-way ANOVA, *n* = 17) and no change in 10–90% rise time (Figures 3F,G, grey bars). These effects on decay and rise times were found not to differ between ST034307 and control experiments (*p* > 0.05, 2-way ANOVA).

### Effect of ST034307 on pacemaker activity in isolated sino-atrial node myocytes

**FIGURE 4:**
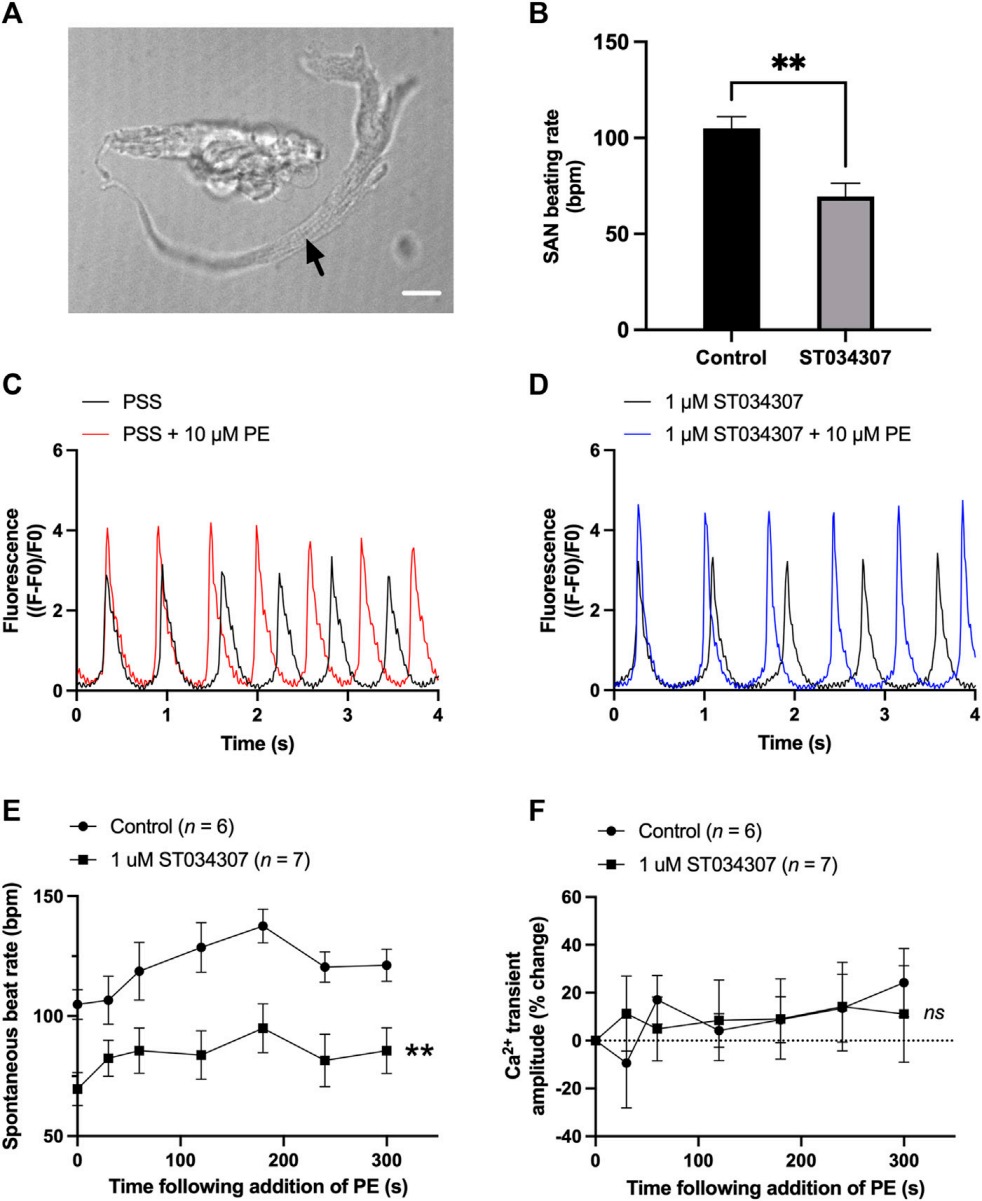
1 µM ST034307 inhibits the basal spontaneous beating rate and response to PE of isolated guinea pig SAN cells. **(A)** Brightfield image to show example of an isolated guinea pig SAN cell as indicated by black arrow. Scale bar represents 10 µm. **(B)** Effect of 1 µM ST034307 on the basal SAN cell Ca^2+^ transient amplitude before addition of PE. **(C,D)** Representative 4 s recordings of changes in fluorescence (expressed as (F-F0)/F0) from spontaneously beating guinea pig SAN cells loaded with Fluo-5F-AM in PSS **(C)** or in PSS +1 µM ST034307 **(D)**. Black and red/blue traces represent recordings before and 5 min after addition of 10 μM PE, respectively. Traces have been aligned according to the peak of the first transient. **(E)** Effect of 10 μM PE on the spontaneous beating rate of isolated SAN cells in the presence (squares) and absence (circles) of 1 µM ST034307. **(F)** Effect of 10 μM PE on the Ca^2+^ transient amplitude recorded from isolated SAN cells in the presence (squares) an absence (circles) of 1 µM ST034307. Time in E and F represents time following addition of PE to the perfusion solution with 0s representing levels in absence of PE. All experiments were carried out at 35 ± 2°C. Data are represented as mean ± SEM. Data in **(B)** were analysed using unpaired t-test. Data in **(E)** were analysed using two-way, repeated measures ANOVA followed by Šídák’s multiple comparisons test. Data in **(F)** were analysed using mixed effects analysis followed by Šídák’s multiple comparisons test; *ns* = not significant; ** = *p* < 0.01; *n* (control) = 6; *n* (ST034307) = 7; *N* = 5 animals.

To assess the contribution of AC1 to rate and calcium signalling in SAN cells, the response to PE under control conditions and during AC1 inhibition with ST034307 was measured in isolated, spontaneously firing guinea pig SAN myocytes loaded with Fluo-5F-AM. Active, spontaneously beating SAN cells were identified based on morphology as indicated by the black arrow in Figure 4A. Under control conditions, fluorophore-loaded SAN myocytes superfused with PSS at 35°C spontaneously contracted at a rate of 104.9 ± 6.2 bpm (*n* = 6, Figure 4B, black bar). Although this rate is below the normal physiological rate for guinea pig SAN, lower rates are expected following the loading of cells with a calcium fluorophore (Rigg and Terrar, 1996). Inclusion of 1 µM ST034307 in the perfusion solution resulted in a mean beating rate of 69.6 ± 6.8 bpm (*n* = 7, Figure 4B, grey bar) representing a significantly lower (0.34-fold) beat rate compared to control (*p* = 0.003, unpaired t-test). Superfusion of 10 μM PE led to a gradual increase in both calcium transient amplitude as well as the peak-to-peak firing rate over the course of 5 min as shown by the example traces in Figures 4C,D. On addition of 10 μM PE, the beating rate of SAN cells in the absence of ST034307 rose from 104.9 ± 6.2 bpm to a peak of 137.5 ± 7.0 bpm after 3 min (*p* < 0.05), before decreasing to a plateau, corresponding to 121.2 ± 6.7 at 5 min (Figure 4E, *n* = 6), potentially caused by desensitisation of α-adrenoceptors (Hiremath et al., 1991; Arce et al., 2017). In the presence of ST034307, beating rate increased to 94.9 ± 10.2 after 60s (Figure 4E, *n* = 7, *p* < 0.05). Overall, 1 µM ST034307 was found to significantly inhibit the rise in SAN cell spontaneous beating rate in response to superfusion with 10 μM PE (*p* = 0.008, mixed-effects analysis) (Figure 4E). Qualitatively, this increase was no longer gradual but plateaued rapidly, being complete by the time of the 30 s recordings (Figure 4E). Although qualitatively an increase in Ca^2+^ transient amplitude was observed in response to 10 μM PE, as shown by the representative traces in Figures 4C,D, this increase was not found to be significant, as shown in Figure 4F (*p* = 0.10, mixed-effects analysis). This may be a consequence of the increased firing rate in response to PE, which would be expected to limit increases in calcium transient. However, in the presence of ST034307, mean Ca^2+^ transient amplitude was found to be consistently lower in the presence of ST034307 after 60 s perfusion with PE (Figure 4F).

## Discussion

There is increasing evidence that activation of the IP_3_ signalling pathway in atrial cells leads to the downstream activation of membrane bound Ca^2+^-sensitive adenylyl cyclases, principally AC1 and AC8 (Mattick et al., 2007; Burton and Terrar, 2021; Capel et al., 2021). Recently published work has demonstrated that inhibition of IP_3_R using 2-APB, and non-specific inhibition of ACs using MDL-12,330A, prevents the rise in spontaneous beating rate observed in response to the α-adrenergic agonist PE in intact mouse right atria, as well as the rise in Ca^2+^ transient amplitude resulting from the intracellular release of caged-IP_3_ in isolated guinea pig atrial myocytes (Capel et al., 2021). The purpose of the current study was therefore to investigate the effect of pharmacological modulation of AC1, using the drug ST034307, to determine if AC1 may play a role in the downstream effects of IP_3_ signalling in cardiac tissue. Taken together, the data presented here suggest that AC1 plays a role in rate regulation at the SAN but is less important in determining inotropic responses.

### The role of AC1 and IP_3_ signalling in the heart

As shown in Figure 1A, 1 µM ST034307 was found to significantly inhibit the increase in spontaneous beating rate of intact right atrial preparations. In contrast, ST034307 did not have a significant effect on the positive inotropic response to PE in paced murine left atria (Figure 1B) or potentiation of the Ca^2+^ transient in isolated guinea-pig atrial myocytes (Figure 3). The simplest explanation for this observation is that AC1 is not involved or minimally contributes to the downstream effect of IP_3_ signalling in non-SAN atrial myocytes but plays a more dominant role in the regulation of SAN pacemaker cells. Such an observation would be consistent with the observation that AC1 is preferentially expressed in the SAN and able to regulate I_f_ (Mattick et al., 2007).

Our immunohistochemistry work suggests that AC1 and IP_3_R2 are located within close proximity in guinea-pig atrial and SAN myocytes (Figure 2). Colocalization in SAN was higher than that observed in right atrial cells (Supplementary Figure S2). Although higher resolution studies would be required to establish colocalization within the level required to form a localised signalling domain, the proximity of IP_3_R2 and AC1 in both SAN and atrial cells demonstrated in Figure 2 and Supplementary Figure S2 raises the possibility that Ca^2+^ released *via* IP_3_R2 may influence the activity of AC1 in the SAN. Due to the relatively low resolution of fluorescent immunocytochemistry, the exact localisation of AC1 in cardiac cells, which may be dynamic, remains unclear. In both SAN (Figure 2A) and atrial cells (Supplementary Figure S2), expression of both AC1 and IP_3_R2 was concentrated most strongly within the periphery of the cells, however it was also seen along the striations, corresponding to t-tubules. AC1 has previously been found to be preferentially found in caveolae in rabbit SAN cells (Younes et al., 2008), while in mouse SAN cells, IP_3_R have been found to be in terminal SR in close proximity to the plasma membrane (Ju et al., 2011). These patterns of expression appear to coincide with our own observations for expression in guinea pig atrial and SAN cells (Figure 2). Previous work has suggested that AC1 might be located close to but not at the surface membrane, though still within reach of Ca^2+^ released *via* IP_3_R (Collins and Terrar, 2012; and see Burton and Terrar, 2021). Higher resolution EM work will be required in the future to provide more information on the precise location of AC1.

Ca^2+^-sensitive ACs are implicated in atrial IP_3_ signalling since BAPTA, MDL-12,330A and W-7 (calmodulin inhibitor) all abolish the effects of PE on I_CaL_ (Wang et al., 2005). It is possible that IP_3_ mediated Ca^2+^ release can lead indirectly to AC1 activation via triggering other Ca^2+^ signals in different regions of the cells. One hypothesis that has been proposed is that local SR Ca^2+^ release from IP_3_R leads to a localized elevation in [Ca^2+^] at the ryanodine receptor, leading to amplification of RyR Ca^2+^ release and activation of LTCC (Lipp et al., 2000; Liang et al., 2009). However, the abolition of response to exogenously applied IP_3_ in the presence of MDL appears to rule out this possibility, as this pathway would be expected to remain following inhibition of ACs (Capel et al., 2021). Another possibility however is that IP_3_R Ca^2+^ release triggers activation of AC1 indirectly, for example *via* amplification of store operated Ca^2+^ entry (SOCE). In HEK293 cells, AC1 and AC8 are significantly activated by SOCE (Fagan et al., 1996) and SOCE is known to occur in close proximity to IP_3_Rs (Sampieri et al., 2018). Furthermore, it has been shown that AC8 interacts directly with Orai1, the pore forming subunit of SOCE channels (Willoughby et al., 2012), and in HeLa cells, activation of IP_3_R clusters tethered below the plasma membrane by the KRas-induced actin-interacting protein (KRAP) leads to localised depletion of ER Ca^2+^, which in turn leads to SOCE via the activation of stromal interaction molecule 1 (STIM1) (Thillaiappan et al., 2021). Interestingly, in isolated mouse SAN cells, the SOCE inhibitor SKF-9665 inhibited Ca^2+^ influx in SAN in response to pharmacological SR unloading and reduced the spontaneous rate by 27% in these conditions (Ju et al., 2007). It remains to be explored whether this finding involves Ca^2+^-activated adenylyl cyclases. The roles of IP_3_ and activation of SOCE in cardiac cells, and the potential for downstream regulation of AC activity, therefore warrants future investigation.

### The role of AC1 and IP_3_ signalling in cardiac pacemaker activity

Pacemaker activity in mouse SAN cells can undergo modulation by both IP_3_ agonists and antagonists, and this modulation can be abolished following IP_3_R2 knock-out, demonstrating that IP_3_ signalling can play a role in regulating pacemaker activity (Ju et al., 2011). In addition, IP_3_ has been shown to induce Ca^2+^ sparks in close proximity to the surface membrane in pacemaker cells, and it has been suggested that this may lead to modulation of inward Na^+^/Ca^2+^ exchange current or activation of alternative Ca^2+^ dependent currents (Ju et al., 2012). Both IP_3_R2 (Ju et al., 2011) and AC1 (Mattick et al., 2007; Younes et al., 2008) are expressed in the SAN (Figure 2), and previous studies have shown potential for activation of Ca^2+^-sensitive adenylyl cyclases downstream of IP_3_R Ca^2+^ release in SAN cells as well as in non-pacemaker cells (Mattick et al., 2007; Ju et al., 2012). These previous observations support a role for the Ca^2+^-activated adenylyl cyclases AC1 and AC8 in atrial and SAN IP_3_ signalling, however the involvement of other adenylyl cyclases cannot be ruled out based on these data alone.

In our experiments, ST034307 was found to significantly reduce the positive chronotropic effect of PE in mouse right atria, reducing the maximal response to PE without changing EC_50_ (Figure 1A), suggesting that the effects of ST034307 are via inhibition of a target within the IP_3_ signalling pathway. However, the effects of PE on inotropy in the left atria were unaffected by ST034307 (Figure 1B). Similarly, ST034307 reduced the spontaneous Ca^2+^ transient firing rate in isolated guinea-pig SAN cells (Figure 4B), as well as the response to PE in SAN cells (Figure 4E) but did not inhibit increases in Ca^2+^ transient amplitude in response to PE in either atrial (Figure 3) or SAN cells (Figure 4). AC1 is implicated as being directly involved in the positive chronotropic effect of the IP_3_ signalling pathway since application of the non-specific AC inhibitor MDL-12,330A abolishes the positive chronotropic response to PE in the absence of β-adrenergic signalling (Capel et al., 2021), thus supporting that cAMP production by ACs is involved in the chronotropic response to IP_3_R activation. Consistent with this hypothesis, AC1 and AC8 are present in the SAN plasma membrane and either or both isoforms potentiate the pacemaker current (Mattick et al., 2007). Furthermore, both AC1 (Younes et al., 2008) and IP_3_R2 localise in close proximity to caveolae in SAN cells (Barbuti et al., 2004; Ju et al., 2011).

Interestingly, in intact, spontaneously beating right atria, we did not observe a decrease in beating rate on addition of ST034307 before addition of phenylephrine (Supplementary Figure S1). Based on the observation that basal rate was decreased in isolated guinea pig SAN cells (Figure 4B), it would be expected that a decrease would also be observed in the intact SAN on addition of ST034307. In previous work, non-selective inhibition of adenylyl cyclase using MDL-12330 has been shown to decrease basal activity in mouse right atria in the presence of β-adrenergic inhibition (Capel et al., 2021). In the present study, the lack of inhibition in basal activity in the intact mouse right atria following inhibition of AC1 may reflect compensation by the calcium sensitive adenylyl cyclase AC8.

The specificity of cAMP signalling is known to rely on localisation within micro- (Zaccolo and Pozzan, 2002) and nano-domains (Surdo et al., 2017). Traditionally, cAMP has been thought to act primarily *via* protein kinase A (PKA) (Walsh et al., 1968; Krebs and Beavo, 1979), and cyclic nucleotide-gated ion channels (Fesenko et al., 1985) to influence cardiomyocyte contractile sensitivity as well as regulating L-type Ca^2+^ channel (LTCC) activity (Chen-Izu et al., 2000; Harvey and Clancy, 2021). However more recent work has demonstrated that cAMP may also act via exchange proteins directly activated by cAMP (EPACs) (de Rooij et al., 1998), as well as “Popeye domain” containing proteins (Brand, 2005; Zaccolo et al., 2021). The downstream effects of cAMP signalling in cardiomyocytes can therefore influence a wide range of cellular processes, including gene expression and cell morphology, in addition to electrical and contractile activity (Harvey and Clancy, 2021). This heterogeneity in function is the result of spatial confinement and localised compartmentalisation of cAMP signalling (Zaccolo and Pozzan, 2002; Zaccolo et al., 2021). The existence of a compartmentalised signalling domain involving both AC1 and IP_3_R2 in SAN cells could explain the results demonstrated in the current study as well as previous observations (Capel et al., 2021). Such localisation would explain why the specificity of this Ca^2+^ signal is not lost despite constant global Ca^2+^ transients within SAN cells and may also explain why inhibition of AC1 by ST034307 inhibits beating rate in the SAN without causing an inhibition of calcium transients, which result from downstream release of calcium via RyR, in atrial and SAN cells (Figures 3, 4). Moreover, since AC1 regulation by Ca^2+^ is biphasic (Fagan et al., 1996), it is possible that IP_3_ induced stimulation of AC1 only occurs after cytosolic Ca^2+^ and the membrane potential have decreased, meaning this additional cAMP is likely only produced during the early and late phase of diastole allowing stimulation of I_f_ by AC1 at the correct time point. Our findings suggest that within SAN cells, Ca^2+^ released from IP_3_R can activate either directly or indirectly AC1 and that this can modulate both basal and stimulated pacemaker mechanisms. Such a mechanism would be comparable but independent of that by which the release of Ca^2+^ from RyR (Rigg and Terrar, 1996; Bogdanov et al., 2001), or Ca^2+^ influx via the L-type (Mangoni et al., 2003; Jones et al., 2007) or T-type Ca^2+^ channels (Huser et al., 1996) regulates basal pacemaker activity.

In the present study, β-adrenergic stimulation was excluded by the inhibition of β-adrenergic receptors using metoprolol, however calcium release following β-adrenergic stimulation could also be expected to result in activation of AC1, which has been shown to respond to Ca^2+^ at concentrations corresponding to the full physiological range (Younes et al., 2008). It has been previously shown however that basal cAMP in the SAN is maintained by Ca^2+^-sensitive ACs and that this process is sensitive to cytosolic Ca^2+^ buffering using BAPTA, whereas changes in cAMP in response to β-adrenergic stimulation are not (Younes et al., 2008). These previous observations, together with our current observations in the absence of β-adrenergic stimulation provide further indirect evidence that the regulation of calcium sensitive ACs in the SAN occurs independently of β-adrenergic stimulation, likely due to the compartmentalisation of calcium signalling with these cells, which would result from the combination of close proximity of IP_3_R2 and AC1, coupled with the limitation of cAMP diffusion by PDEs (Zaccolo and Pozzan, 2002; Surdo et al., 2017).

Of note, 1 µM ST034307 did not abolish the response to PE in right atrial tissue (Figure 2A), although based on the reported IC_50_ of 2.3 µM (Brust et al., 2017), this dose of ST may have been insufficient to cause maximal AC1 inhibition. Whilst our findings suggest a role for AC1 downstream of IP_3_ mediated Ca^2+^ release, in the absence of a specific AC8 inhibitor, our results cannot rule out the possibility that AC8 is also involved. At the time of writing no such specific AC8 inhibitor is commercially available.

### Clinical relevance

Abnormal Ca^2+^ signalling underlies the pathology of many forms of cardiac disorders and arrythmias, including AF (Landstrom et al., 2017). Current rate control medication for diseases such as heart failure and AF target either β-adrenergic signalling, Na^+^/K^+^-ATPase, or I_f_, while few selective pharmacological treatments exist for sinus node dysfunction. Expression of IP_3_R2 in atrial cardiomyocytes is known to be six times greater in atrial myocytes compared to ventricular myocytes (Lipp et al., 2000), and IP_3_R2 is the predominant isoform in the SAN, showing similar expression levels to right atrial tissue (Ju et al., 2011). Expression of IP_3_R is known to be upregulated in human patients with chronic AF (Yamda et al., 2001) as well as the canine AF model (Zhao et al., 2007). The IP_3_ signalling pathway has therefore been identified as a potential atrial-specific target for the treatment of AF (Tinker et al., 2016), and the existence of a downstream AC1 dependent pathway in atrial and SAN tissue could therefore hold promise for the development of pharmacological interactions that selectively target cardiac AC1. Despite this, a definitive link between IP_3_R Ca^2+^ release, AC1 and pathogenesis of AF is yet to be shown and further research investigating this link is required. Additionally, sinus node dysfunction (or sick sinus syndrome) comprises a group of progressive non-curable diseases where the heart rate is inappropriately bradycardic or tachycardic, resulting in increased morbidity rates (Alonso et al., 2014). In familial sinus node dysfunction, multiple different mutated proteins have been implicated including key Ca^2+^ handling proteins such as RyR2, calsequestrin and Cav1.3, alongside HCN4 (Wallace et al., 2021). The crucial importance of Ca^2+^ handling and signalling to pacemaker function and apparent importance in sinus node dysfunction makes this an important potential target for the modulation of pacemaker activity in patients with sinus node dysfunction as well as patients with heart failure and AF. However, a directed approach to finely modulate SAN Ca^2+^ signalling directly is as yet to be described.

### Limitations of this study

ST034307 is reported to be highly selective for AC1 at lower concentrations and does not inhibit other AC isoforms at concentrations below 30 µM (Brust et al., 2017). Despite this, we found that in the presence of 10 µM ST034307 heart rate was dramatically reduced compared to baseline on addition of PE at concentrations over 10 μM, and as such we used a lower concentration of 1 µM ST034307. Although this concentration is below the reported IC_50_ of 2.3 µM reported by Brust et al*.* (2017) 1 µM ST034307 would still be expected to result in around 40% occupancy based on the inhibition of the effect of A-23187 in HEK cells reported previously (Brust et al., 2017). Despite the use of this lower concentration, chromones have a widely documented biological activity in a range of biological settings (Gaspar et al., 2014), and the possibility of off target effects of ST034307 cannot be eliminated. At higher concentrations (≥30 µM), ST034307 shows potentiation of AC2, and moderate potentiation of AC5/6, however these observations have not been reported at the lower concentration (1 µM) used in the present study (Brust et al., 2017). Furthermore, the net effect of ST034307 in the SAN is expected to favour AC1 inhibition due to the higher expression (Mattick et al., 2007; Younes et al., 2008) and activity of AC1 (Younes et al., 2008) compared to AC2, AC5 and AC6 within SAN cells. Of note, entry of the structure of ST034307 into the SwissTargetPrediction tool (Gfeller et al., 2014) did not identify a high probability of interactions in either mouse or human and failed to identify any potential interactions with ion channels. For mouse, the SwissTargetPrediction tool identified, as the highest likelihood, only a 0.06% probability of interaction with histone deacetylase 6 and 8, and D-amino-acid oxidase with no other predicted interactions. In human, in addition to those interactions also identified for mouse, a 0.06% probability of interaction was also identified for quinone reductase 2 and histone deacetylase 2. To validate this low prediction of off-target interactions, future investigations using the genetic knockdown or knockout of AC1 in cardiac tissue, including the SAN, would provide a more comprehensive understanding of the role played by AC1 downstream of IP_3_ signalling in the heart.

By using intact tissue and cells, the data presented here provide indirect evidence that modulation of AC1 regulates SAN pacemaker activity downstream of α-adrenergic stimulation. However, additional electrophysiological data would be required to demonstrate this influence directly, and further experiments are required to determine the mechanism by which AC1 activity regulates pacemaker function. Despite this however, by using intact cells, the pharmacological approach used in the present study avoids the disruption of intracellular signalling that would be expected using more cellular invasive techniques such as whole-cell patch clamp.

## Conclusion

The present study highlights a role for the Ca^2+^-dependent AC isoform AC1 in influencing cardiac pacemaker activity, both at the level of the isolated SAN cell as well as at the level of the intact beating right atria. The most likely explanation for the blunting of the positive chronotropic response of the SAN to PE is inhibition of AC1 by ST034307. Moreover, the cause of the divergent effects of 1 µM ST034307 between the SAN and atrial myocytes merits further investigation of the mechanisms regulating both atrial and SAN IP_3_ signalling.

Overall, this study provides additional support for the existence of an IP_3_ → AC1 → cAMP signalling pathway, and a role for this pathway in the regulation of SAN pacemaker activity in response to α-adrenergic signalling. The findings presented support a role for AC1 downstream of IP_3_-mediated Ca^2+^ release, providing a new example of how crosstalk between Ca^2+^ and cAMP signalling is involved in regulating SAN pacemaker activity. These data add to the already published mechanism of crosstalk between Ca^2+^ and cAMP signalling within the SAN, with Ca^2+^ already having been shown to control basal cAMP levels and pacemaker activity (Mattick et al., 2007; Younes et al., 2008), and provide further support for previous work identifying a link between IP_3_ signalling and the downstream activation of Ca^2+^-dependent ACs (Capel et al., 2021). Furthermore, the concept that SAN IP_3_ signalling, and automaticity can be targeted through cyclic nucleotide signalling suggests further investigation of putative IP_3_-cAMP signalling pathways in cardiac atria may identify novel targets, for example phosphodiesterases, to modulate pacemaker activity and also prevent the triggering of atrial arrhythmias such as AF.

## Data Availability

The raw data supporting the conclusion of this article will be made available by the authors, without undue reservation.

## Supplementary material

SUPPLEMENTARY FIGURE S1 Effect of different combinations of metoprolol (1 μM), ST034307 (1 μM) and PE (10 μM) on mouse right atrial beating rate. Bars represent mean heart rate (bpm) under different conditions as indicated below the x-axis. Significance bars represent comparison of the data using ANOVA followed by Fisher’s LSD test to compare all conditions (non-significant comparisons are not shown) (*n* = 11–14). Data are represented as mean ± SEM.

SUPPLEMENTARY FIGURE S2
**(A)** Representative example of a fixed, isolated guinea pig atrial myocyte (i) immunolabelled for IP_3_R2 (magenta), (ii) AC1 (cyan), (iii) co-immunolabelled for IP_3_R2 (magenta) and AC1 (cyan) and (iv) bright field. **(B)** Intensity plot to show staining intensity along the line shown in **(A)**. **(C,D)** Intensity surface plot showing the distribution of staining of IP_3_R2 [magenta, **(C)**] and AC1 [cyan, **(D)**] for the whole cell, as shown in **(A)** (iii). Scale bars representing 10 µm are indicated in **(A)**. For the purposes of presentation only, red and green channels have been represented as magenta and cyan, respectively.
