# Supplementary figures and images for: Inhibition of adenylyl cyclase 1 by ST034307 inhibits IP3-evoked changes in sino-atrial node beat rate
Source: Front Pharmacol. 2022 Aug 29;13:951897. doi: 10.3389/fphar.2022.951897 (PMC9465815; doi:10.3389/fphar.2022.951897)

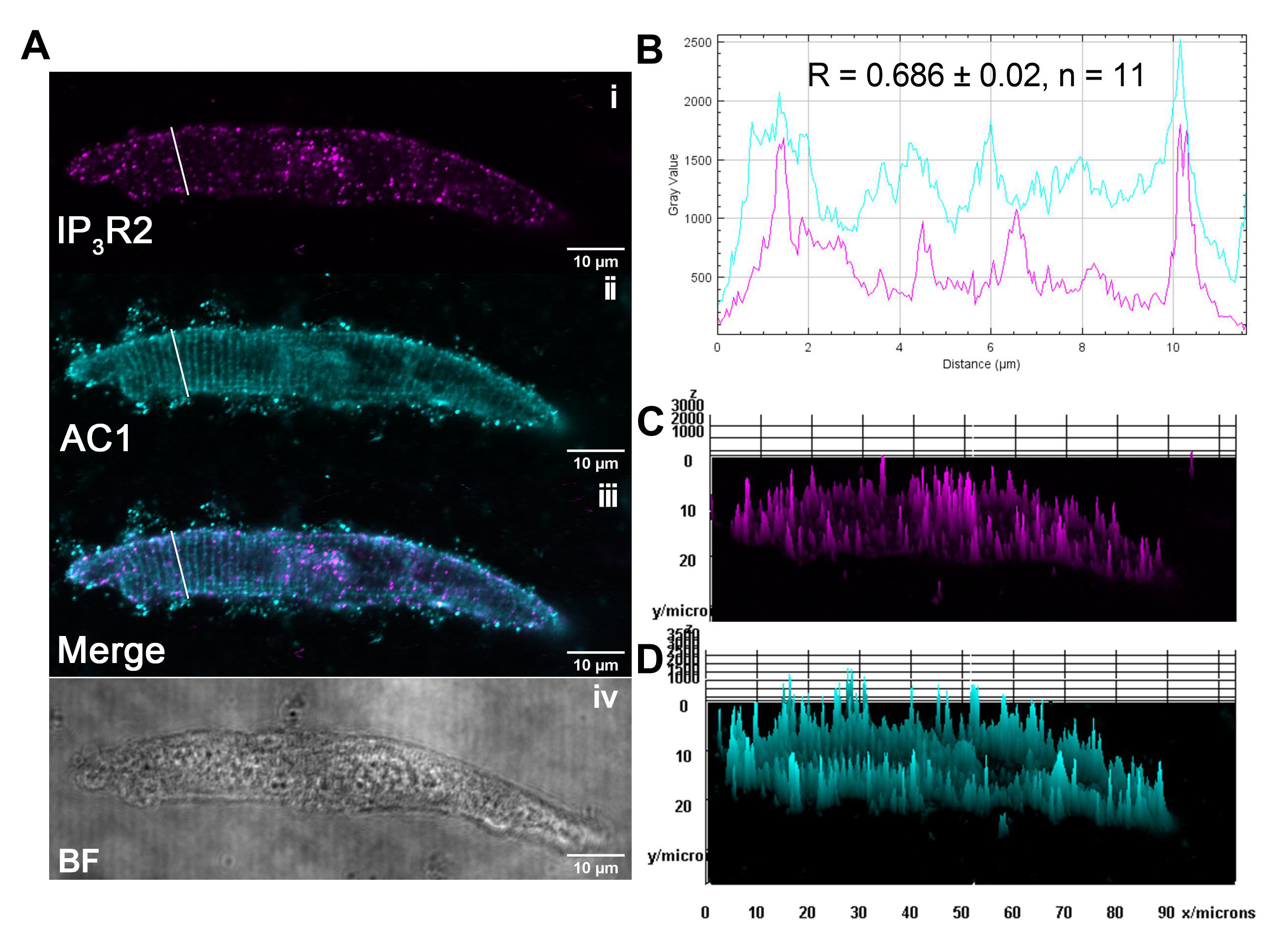

Supplement: Supplementary file 1 [file Image2.TIF]

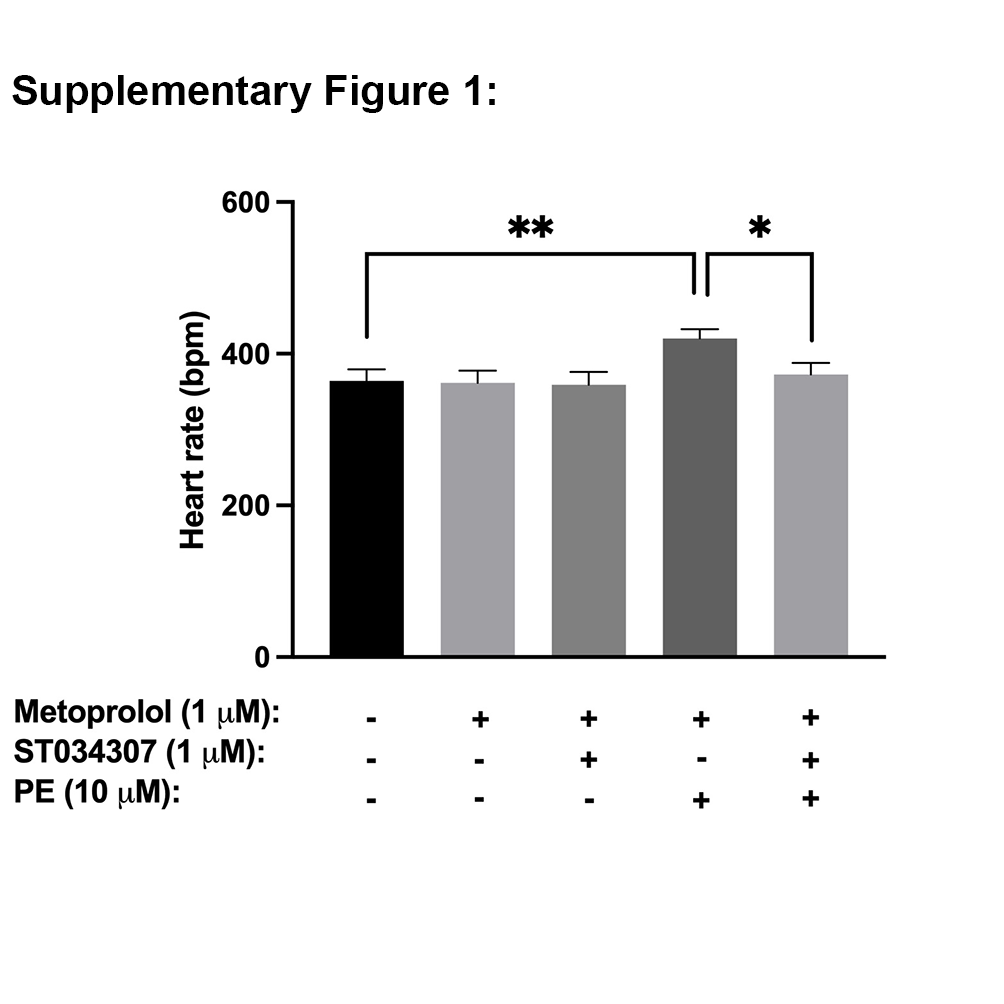

Supplement: Supplementary file 2 [file Image1.TIF]
